# Analytical challenges of glycosaminoglycans at biological interfaces

**DOI:** 10.1007/s00216-021-03705-w

**Published:** 2021-10-14

**Authors:** Gergo Peter Szekeres, Kevin Pagel, Zsuzsanna Heiner

**Affiliations:** 1 Institut für Chemie und Biochemie, Freie Universität Berlin, Arnimallee 22, 14195 Berlin, Germany; 2 Department of Molecular Physics, Fritz-Haber-Institut der Max-Planck-Gesellschaft, Faradayweg 4-6, 14195 Berlin, Germany; 3 School of Analytical Sciences Adlershof, Humboldt-Universität zu Berlin, Albert-Einstein-Straße 5-11, 12489 Berlin, Germany

**Keywords:** Glycosaminoglycans, Lipids, Nonlinear spectroscopy, Sum-frequency generation spectroscopy, Interface/surface analysis

## Abstract

The analysis of glycosaminoglycans (GAGs) is a challenging task due to their high structural heterogeneity, which results in diverse GAG chains with similar chemical properties. Simultaneously, it is of high importance to understand their role and behavior in biological systems. It has been known for decades now that GAGs can interact with lipid molecules and thus contribute to the onset of atherosclerosis, but their interactions at and with biological interfaces, such as the cell membrane, are yet to be revealed. Here, analytical approaches that could yield important knowledge on the GAG-cell membrane interactions as well as the synthetic and analytical advances that make their study possible are discussed. Due to recent developments in laser technology, we particularly focus on nonlinear spectroscopic methods, especially vibrational sum-frequency generation spectroscopy, which has the potential to unravel the structural complexity of heterogeneous biological interfaces in contact with GAGs, in situ and in real time.

**Graphical abstract:**

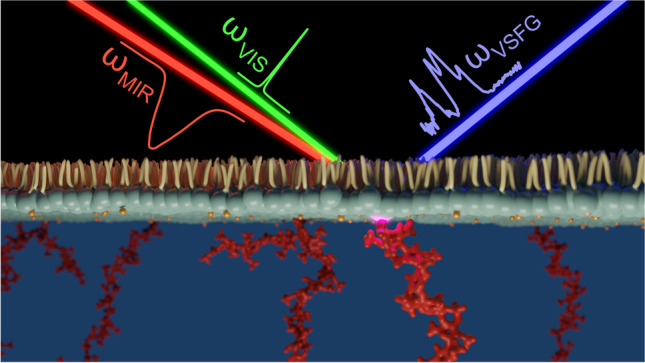

## Analytical challenges of glycosaminoglycans

Glycosaminoglycans (GAGs) are negatively charged, linear polysaccharides composed of repeating disaccharide building blocks containing a hexuronic acid and a hexosamine unit. Their negative charge originates from carboxylate groups in the hexuronic acids and sulfate groups at various positions of the hexuronic acid (2S) and the hexosamine (NS, 4S, 6S) moieties. The varying length of GAGs and their diverse sulfation patterns render their analysis a challenging task due to their high chemical and structural similarity. However, a correct and precise analysis of GAGs is crucial, which became especially apparent in 2008 in the Heparin Adulteration Crisis, in which heparin, a highly sulfated GAG used as an anticoagulant drug, was found to be contaminated with highly sulfated chondroitin sulfate, a structurally different GAG [1]. The administration of the adulterated heparin resulted in nearly a thousand health-related emergencies and deaths.

GAGs are usually located at the cell membrane, in the pericellular and extracellular matrix, and in granules within certain hematopoietic cells. This often means that GAGs are found at biological interfaces and barriers, which allow passage of specific small molecules, while larger molecules are retained at the exterior. These barriers can be covered by a layer called the glycocalyx, which in the past decade has gained increasing interest due to its prominent role in processes such as cancer development or drug delivery through the blood-brain-barrier [2]. Therefore, it is crucial to gain an in-depth knowledge about its constituents and their interaction at biological interfaces.

**Fig. 1:**
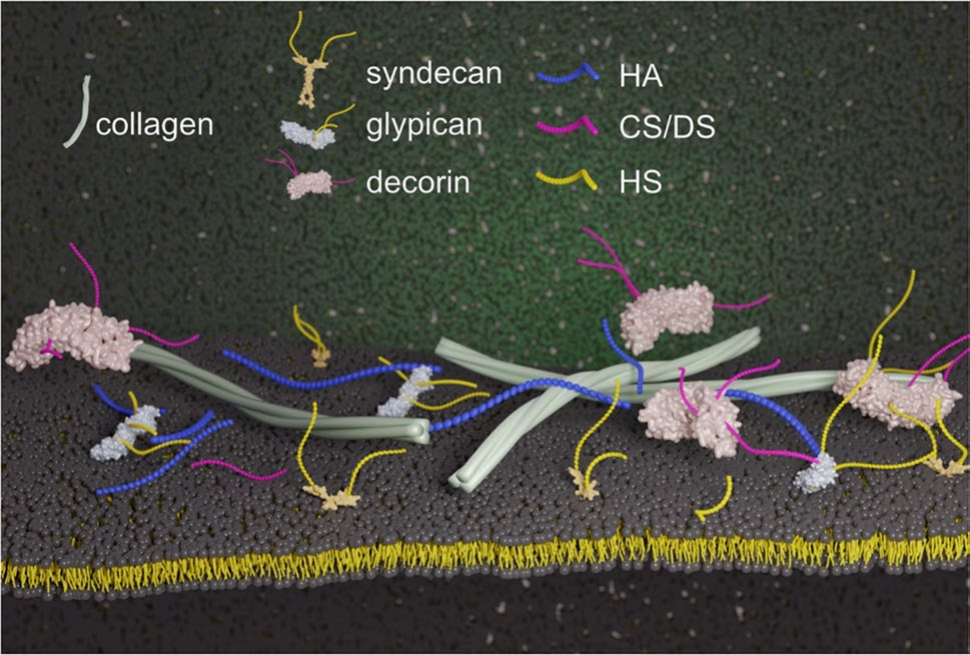
Schematic representation of the extracellular matrix-cell membrane interface, where transmembrane (*e.g.*, syndecan), membrane-bound (*e.g.*, glypican), and free proteoglycans (e.g., decorin), as well as glycosaminoglycans (GAGs) can interact with collagen, membrane proteins, and the lipids of the cell membrane. The GAG chains of hyaluronic acid (HA), chondroitin/dermatan sulfate (CS/DS), and heparan sulfate (HS) are color-coded as blue, magenta, and yellow, respectively

The analytical challenges of GAGs are a direct result of their structural diversity. GAGs are very abundant in the extracellular matrix (ECM) where they appear in free form or covalently attached to proteins as proteoglycans (PGs) (Figure 1). Each PG has one or more serine residues, which each carry post-translationally attached GAG chains that determine the role of the PG in the organism. There are four distinct groups of GAGs: heparin (Hep)/heparan sulfate (HS), chondroitin sulfate (CS)/dermatan sulfate (DS), keratan sulfate (KS), and hyaluronic acid (HA). Out of these four groups, Hep/HS and CS/DS are the most complicated molecules, but also those that readily participate in a plethora of biological processes. In PGs, Hep/HS and CS/DS are covalently bound to a Ser residue via a xylosylated tetrasaccharide linker, while KS binds to *N-* and *O-*glycans.

## Isolation and synthesis of GAGs

In order to discuss the isolation and synthesis of GAGs, it is first crucial to understand the biological processes via which GAGs are biosynthesized. The biosynthesis of GAGs can be generally divided into two steps after the synthesis of the xylosylated tetrasaccharide linker on the specific Ser residues: the first step is the chain elongation, during which the hexuronic acid and hexosamine units are assembled into a chain [3]. In the second step, a variety of enzymes, such as deacetylases, sulfotransferases, and epimerases modify the chain with the possible removal of the acetyl group and the addition of sulfate groups in different positions in the disaccharide building blocks, thus producing a high variety of GAG molecules [3].

Naturally produced GAG chains, which are usually covalently bound to a proteoglycan, can be isolated either by beta elimination or by fully digesting the core protein of the proteoglycan, where the GAG chain is bound to a Ser residue via a tetrasaccharide linker. Once the protein digestion is complete, the GAG chain can be depolymerized by lyases and hydrolases. The choice of enzyme is crucial as the product of the depolymerization is enzyme-specific. It is possible to produce a wide range of different chain lengths by incomplete depolymerization, which in general falls in the degree of polymerization (dp) range of dp4–dp20. After the digestion and possible depolymerization, the GAGs undergo purification and enrichment, which often involves the use of an anion-exchange column or different chromatography techniques, such as size-exclusion chromatography and reverse-phase ion-pairing liquid chromatography [4].

The abovementioned techniques are useful tools to isolate GAGs from tissues that are rich in proteoglycans. However, the synthesis and selective sulfation of smaller GAG oligosaccharides has also been made possible by automated synthesis relying on specific blocks and the use of protective groups during polymerization [5]. More recently, the chemoenzymatic synthesis of GAGs involving the bacterial fermentation of the polysaccharide backbone and the subsequent sulfation with selective sulfotransferases allows for the higher yield production of GAGs, but this technique is generally used for the synthesis of highly sulfated chains, mostly Hep/HS [6, 7]. In addition, a library of genetically engineered cells which express differently modified GAG structures termed the *GAGome* was first reported in 2018, providing means to study the effects of these molecules under much more controlled circumstances than ever before [8].

## Interactions of GAGs

### GAG-protein interactions

Although GAGs play numerous roles in our body, including those in cancer, inflammation, embryonic development, or neuroplasticity, their interactions with other biomolecules pose as an exciting area of research [9]. GAGs are often found as polyanions at the cell membrane and in the extracellular matrix, which makes them prone to interact with other biomolecules, which carry positive charges or positively charged patches on their surfaces, such as proteins. The study of protein-GAG interactions, termed the *GAG interactome*, has been a hot topic for the bioanalytical community for the past decades [10, 11]. While heparin and its derivatives have long been used in hospitals as anti-coagulants, brought about by their interaction with antithrombin [12], and the role of Hep/HS in inducing cytokine storm has also been emergingly studied [13], the major focus of the scientific community has turned towards GAG-protein interactions in 2020, when it was found that HS plays an active role in the host infection of SARS-CoV-2 [14, 15]. To highlight the importance of protein-GAG interactions, a new database called *MatrixDB* has emerged in 2019 to provide a place to collect experimentally confirmed interactions between proteins and GAGs [16].

### GAG-lipid interactions

The study of GAG-lipid interactions reaches back decades [17–24], when most studies in this area were performed to yield a better understanding on the role of GAGs in atherosclerosis. It was found that if the endothelium of the arteries is damaged, proteoglycans from the ECM can migrate into the arterial intima, where they can associate with low-density lipoproteins via a Ca^2+^ bridge [17]. This complex can then bind other lipophilic molecules, such as cholesterol. The continuous growth of this complex can lead to the formation of a plaque that protrudes into the arterial lumen, which alone can lead to insufficient blood flow; at the same time, the possibility of the rupture of the endothelium and the release of a fragment from this plaque can block the flow higher up the blood stream. By the same analogy, such an interaction has also been postulated to contribute to nanoplaque formation, a crucial step in the development of Alzheimer’s disease [17].

However, there might be another aspect to lipid-GAG interactions. As mentioned above, GAGs are abundant in the peripheries of the cell membrane: they can be found in free form in the ECM, or bound to core proteins in ECM- or membrane-related proteoglycans. Therefore, their direct contact with the lipid moiety of the cell membrane is inevitable. Although, the lipid bilayer carries a neutral net surface charge in an environment with neutral pH, it was found that the headgroups of zwitterionic phospholipids can carry a small net positive surface charge at (near-)neutral pH (7.0–7.4) [18, 19], which can be the basis of interaction with the highly negatively charged GAGs. By discovering the interactions of GAGs with the lipid membrane, and the structure of GAGs at the membrane in situ, it will be possible to discuss important aspects of the role of GAGs and their structural properties in, *e.g.*, drug-cell interactions and pathogen-cell infection mechanisms.

## Analytical approaches for GAG-lipid interactions

In the past, especially before attempts made at producing homogeneous GAG chains with identical sulfation pattern were successful, most studies used dextran sulfate as a model molecule for GAGs. In general, GAG-lipid interactions can be observed by monitoring two parameters: (1) the changes of physical properties as a result of GAG-lipid association or (2) the changes in the structure of the lipid film or lipid vesicles due to the adsorption of GAG chains.

Early experiments relied on less complex instrumentation and studied the existence of interaction between GAGs and lipid vesicles. In such experiments, ultracentrifugation was used to precipitate the insoluble dextran sulfate-lipid complexes [20] or benefited from the drastic change of surface charge due to the association of the polyanion by studying changes in the electrophoretic mobility of the vesicles [21]. Soon after these studies, the importance of ^2^H solid-state NMR increased: by quantitatively assessing the quadrupolar splitting of the α- and β-methylene peaks, researchers became able to assess how dextran sulfate interacts with multi-lamellar lipid vesicles [22]. These studies have been often augmented with other NMR techniques, *e.g.*, magic-angle spinning or nuclear Overhauser-enhanced spectroscopy[23], and solid-state structure characterization methods based on X-ray scattering[23, 24].

## Emerging bioanalytical tools for the characterization of GAG-lipid interactions

In order to develop a better understanding of GAG-lipid interactions, it is crucial to perform experiments at physiologically relevant conditions. While solid-state approaches [22] (*e.g.*, NMR) or gas-phase studies[25] (*e.g.*, IR action spectroscopy) yield crucial structural information about homooligomeric complexes or molecules in isolation, it is challenging to study truly unaltered GAG-lipid interactions as these interactions in the body happen in solution and at the lipid-water interfaces.

An abundantly used analytical tool to study biomolecular changes and interactions under physiologically relevant conditions is fluorescence spectroscopy (*e.g.*, super-resolution fluorescence microscopy, fluorescence lifetime imaging, fluorescence correlation spectroscopy, and two-photon fluorescence). From a physical-chemical point of view, however, the results from fluorescence may easily be biased due to the use of fluorescent labels. On the other hand, vibrational spectroscopic techniques such as Raman scattering and infrared (IR) spectroscopy are label-free, yet non-destructive tools to study the structure and chemical composition of matter. In comparison with vibrational spectroscopy, fluorescence spectroscopy lacks the chemical structural and compositional information, and therefore, it can only be an otherwise invaluable complementary tool to Raman scattering and IR spectroscopy. At the same time, normal Raman scattering and IR spectroscopy have their own limitations. GAG-lipid interactions naturally occur in an aqueous environment, which creates difficulties in IR (micro)spectroscopy experiments. With Raman scattering, homogeneous solutions of, e.g., different GAG molecules can be analyzed, but the obtained results may not be relevant on the cellular or tissue level, where both the GAG chains and the interacting partner, e.g., lipid membrane, are expected to be highly heterogeneous. Further difficulties are caused by the fact that even though a local enrichment of GAGs at the cell membrane is expected (either as free molecules or bound to a proteoglycan), their absolute concentration is still rather low—and often too low for normal Raman experiments. Further work is therefore required to overcome the problems and optimize the methodology to identify GAGs in live cells. Advances in surface-enhanced Raman scattering (SERS) can overcome some of the limitations of normal Raman experiments [26, 27]. SERS is well-known for the complex data sets collected from heterogeneous biomolecular systems, and studying a system with highly heterogeneous, yet chemically similar GAGs, the interpretation of the resulting data would require specialized data processing tools. The different ionic strength and pH in the extra- and intracellular matrices together with the highly negative charge of GAGs make the application of nanoparticles generally challenging. Furthermore, the question how the surface charge and chemical composition at interfaces with GAGs influence the nanoparticle uptake and drug delivery is yet to be answered [2].

When choosing the analytical method, one must also consider the sample type where GAG-lipid interactions could be studied informatively. The biological interfaces at which GAGs are expected to be particularly enriched (*e.g.*, glycocalyx, cell membrane, and ECM) are highly heterogeneous and crowded biomolecular environments; therefore, we must select the simplest, yet still heterogeneous biomolecular system as a model interface to be studied by an analytical technique that provides the most detailed information about, *e.g.*, the GAG-lipid interactions in real time.

When considering a spectroscopic tool for the study of real-time GAG-lipid interactions in a physiologically relevant environment, label-free and interface-selective techniques are needed. Such a tool should be sensitive enough to detect the concentration of the (heterogeneous) analyte far below the millimolar range while working in an aqueous environment with interface selectivity and/or sub-micrometer axial resolution. These challenging requirements are clearly fulfilled best by nonlinear spectroscopic techniques. In the following, nonlinear spectroscopic techniques applicable to study GAG-lipid interactions are discussed in detail and summarized in Table 1.

**Table 1:** Summary of selected nonlinear optical techniques for studying GAGs at biological interfaces. General samples include model membranes, single cells, and tissues

Technique	Information	GAG-lipid interactions	Limitations
Coherent Raman scattering (CARS)	Three-dimensional and temporal chemical imaging based on Raman-active vibrational modes, information of enriched molecules	Enrichment and distribution of GAGs at model/cell membranes with high spatial resolution; possibility of distinguishing proteoglycan GAGs from free GAGs	Not surface-selective, only Raman-active modes (*e.g.*, glycosidic bonds) can be studied
Second-harmonic generation (SHG)	Selective to highly organized macromolecular interfaces; this tool can be coupled with other spectroscopic methods within one instrument	Conformational order and alignment (angular distribution) of GAG chains at model/cell membranes, order-disorder of GAGs at interfaces	No chemical structural information on its own, only informs about the existence of a highly organized molecular system
Vibrational sum-frequency generation (VSFG)	Interface-selective vibrational information, no signal contribution from bulk, ordered interface/monolayer is required, vibrational modes that are both IR- and Raman-active are detected	Conformational order, absolute orientation of specific molecular groups at the interface, and secondary/tertiary structure of GAG chains at the model/cell membrane, real time and in situ interaction of GAGs with other biomolecules	High orientational ordering of functional groups is required, limitations in sample preparation (*e.g.*, non-supported lipid bilayers are not possible)

### Coherent Raman scattering spectroscopy

Coherent Raman scattering spectroscopy is a nonlinear technique based on Raman-active vibrational modes, which has evolved into two different techniques: coherent anti-Stokes Raman scattering (CARS) and stimulated Raman scattering (SRS). Both methods depend on a pump laser and a Stokes laser. The former is generally at a fixed wavelength while the latter is tuned to a frequency that is the difference of the pump frequency and the frequency of the molecular vibration of interest. These two laser beams are matched in the focus in both space and time, leading to a vibrational coherence, *i.e.*, a vibrational population with phase-matched vibrations. In SRS, only the pump and the Stokes beams are used to generate the SRS signal, which is detected either as an increase in the measured intensity at the Stokes wavelength (stimulated Raman gain) or as an intensity loss at the pump wavelength (stimulated Raman loss). In CARS, also a third so-called probe beam is used, which excites the aforementioned vibrational population to another virtual vibrational state, from which the molecules relax back to the ground state, generating photons that are blue-shifted (*e.g.*, to the anti-Stokes side of the spectrum) from the probe laser frequency by the frequency of the resonant molecular vibration. While coherent Raman scattering is not interface-selective, it is still a promising candidate to follow the assembly of lipids and GAGs [28], and its ability to provide three-dimensional information with spatial resolution at the order of 100 nm makes it an important tool [29]. These benefits have already been demonstrated when polysaccharide-protein interactions were studied with ^19^F-labeled GAGs under physiological conditions using CARS [30]. The CARS microscopic analysis of GAG-lipid interactions in more complex samples, *e.g.*, single cells or tissues can provide real-time three-dimensional chemical and compositional information about the enrichment of GAGs at the cell membrane.

### Second-harmonic generation spectroscopy

Second-harmonic generation (SHG) spectroscopy is a second-order nonlinear optical tool, where high-intensity, femtosecond laser pulses at frequency *ω* are focused on the sample. Due to the induced second-order material polarization, two photons with the same *ω* frequency interact with the sample and a new photon is generated at the frequency of 2*ω*. SHG is only generated in non-centrosymmetric media, which is fulfilled by highly ordered biomolecular structures. Furthermore, the symmetry is broken at any interface; therefore, molecular monolayers and their dynamics could be visualized by the surface-SHG technique. For example, real-time changes in protein conformation under dynamic conditions were recently studied in the presence of ligands and small molecules [31]. The biggest drawback of SHG is certainly that it generally does not provide structural information, but the technique allows for studying the orientation, conformation, symmetry, and disorder of biological macromolecules (*e.g.*, collagen, myosin, tubulin, cellulose, and starch) and their assemblies in cells and tissues [32]. Furthermore, it is technically relatively straightforward to combine SHG imaging with other techniques such as two-photon fluorescence [33] or CARS microscopy for the collection of three-dimensional structural information in whole cells or tissues [28]. SHG can be useful for the simple determination of the higher order organization, conformation, and order-disorder of GAG chains at the lipid membrane even in real time. When SHG multiplexed with other microscopic techniques, it can provide information about both the interaction and the organization of GAG chains.

### Vibrational sum-frequency generation spectroscopy

**Fig. 2:**
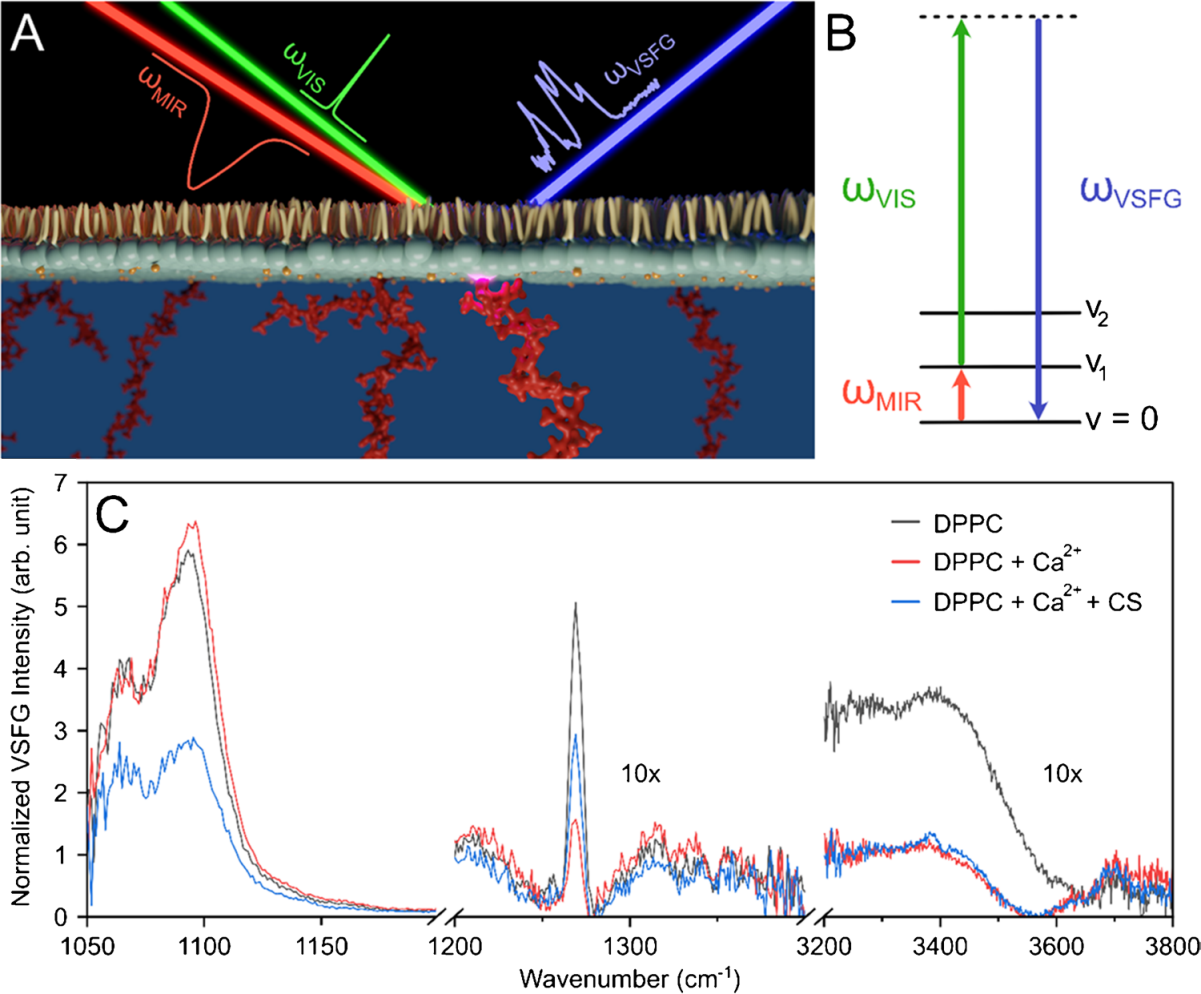
**a** Schematic representation of a VSFG experiment on a lipid monolayer-GAG system **b** with the Jablonski diagram of the VSFG process. **c** Average VSFG spectrum of a monolayer of the phospholipid, dipalmitoylphosphatidylcholine (DPPC), and its interaction with CS in the presence of Ca^2+^ ions at physiologically relevant concentrations. Figure adapted from ref. [39]

Fundamental understanding of the function-structure relations at biological interfaces and biological barriers is crucial where the local microenvironment and the intermolecular interactions can dictate and strongly modify this relation. Vibrational sum-frequency generation (VSFG) spectroscopy is an inherently interface-sensitive analytical tool that has a significant promise for studying the chemical structure, composition, and dynamics of molecules in situ and real time in a label-free manner at such interfaces. Similar to SHG, VSFG spectroscopy is also a second-order nonlinear method. The two incoming electromagnetic fields induce second-order material polarization, resulting in a new photon based on the sum-frequency generation process. Since centrosymmetry is broken at interfaces, the detection and chemical analysis of ordered molecular monolayers become possible even at very low surface coverages. During a broadband VSFG measurement, a narrowband, picosecond visible laser pulses (*ω*_VIS_) are temporally and spatially overlapping with a broadband, femtosecond mid-infrared laser pulses (*ω*_MIR_) at the surface (Fig. 2a and b). The *ω*_MIR_ is resonant with the vibrational mode(s) of the investigated molecule(s). Through the sum-frequency generation process, the molecular vibrational fingerprint, generated by the *ω*_MIR_, is up-converted with the help of the *ω*_VIS_ to the visible spectral range (Fig 2b). The resulting VSFG spectra can be collected with a spectrometer equipped with a CCD. Applying different polarization combinations for each electromagnetic field involved in the SFG process (*e.g.*, *ppp* (or *ssp*), meaning *p*(*s*) for VSFG, *p*(*s*) for visible, and *p*(*p*) for mid-infrared), the amplitudes, frequencies, and spectral widths of the given molecular vibrations can be determined. This polarization-sensitive nature of the technique, together with its interface specificity, enables the identification of molecular structures and orientations of chiral and achiral molecules and molecular groups at interfaces. The method can also work at any interfaces, such as air-water, oil-water, or buried interfaces, highlighting the application of VSFG spectroscopy for the investigation of bio-interfaces. Thanks to the second-order SFG process, vibrational modes that are both IR- and Raman-active can be visualized.

In the last two decades, interfacial water structure and the orientation of lipid monolayers and asymmetric bilayers were thoroughly investigated by using achiral VSFG. On the one hand, this made it possible to understand the structural behavior of lipids during the hydration and their interaction with other biologically relevant molecules such as ions, charged small molecules, peptides, and proteins [34]. On the other hand, these studies helped to establish biologically relevant membrane model systems of different complexity for investigating real-time interactions at model membrane interfaces (*e.g.*, lung surfactants) where the membrane fluidity and rigidity were monitored via the alkyl chain order. Due to the vast number of hydroxyl groups in GAGs and their different chemical environment as opposed to water molecules, changes in the hydration layer can be monitored simultaneously with changes in the chemical environment of GAGs at the interface.

In the past 20 years, studying buried polymer interfaces in situ by VSFG spectroscopy was also a focus in modern polymer science and chemical surface engineering, since the method gives interface-related structural information [35]. From the very beginning of the application of VSFG spectroscopy, the polymer-water interface was the most studied model system. The gained knowledge helped to design more hydrophilic and biocompatible polymers with favorable interfacial structure and composition for, *e.g.*, body implants or drug delivery [36, 37]. All of these experiments conducted at aqueous interfaces will push forward the development of biocompatible and biodegradable polymers for biomedical and food sciences, creating a possibility to revolutionize our future. The development and optimization of analytical tools at such buried interfaces along the way will make it feasible to investigate the structurally highly heterogeneous GAGs at aqueous interfaces as well. For example, in a more recent study, the molecular structure, solvent interaction, and surface dynamics of hyaluronic acid were monitored at the air-water interface [38].

Many important biological molecules such as sugars, amino acids, or nucleic acids have one or more chiral centers. By condensation, these building blocks assemble into larger polysaccharides, proteins, or oligonucleotides, which in turn fold into chiral higher order structures. The development of chiral VSFG spectroscopy has revealed the secondary, tertiary, and higher order structures and orientations of various peptides and proteins in situ and real time [40]. These studies were conducted mainly at the air-water or even at a model membrane interface, allowing to study the folding-misfolding and aggregation of peptides and proteins and following their dynamics at a model membrane interface without dealing with the background signal of the membrane [41]. The advances in investigating the structure of proteins at the liquid, membrane, mineral, and synthetic interfaces were summarized recently highlighting the potential of VSFG spectroscopy in following the molecular-level spatiotemporal evolutions at bio-interfaces [42].

Recent developments in laser technology made it possible to increase the laser repetition rate employed in VSFG spectrometers by two orders of magnitude to 100 kHz [43, 44], which led to a drastic increase in sensitivity and a shorter acquisition time (≤ 10 s) for solid-supported heterogeneous phospholipid monolayers and bilayers [45, 46]. This opens up a plethora of possibilities to study heterogeneous interfaces with increasing complexity in the future. The high sensitivity of the 100-kHz VSFG spectroscopy makes the study of molecular structural changes in, *e.g.*, proteoglycans accessible upon their interaction with other molecules or saccharides with lipids [47].

In a proof-of-concept experiment series, we recently demonstrated that it is possible to study lipid-GAG interactions at the air-liquid interface within a DPPC monolayer on aqueous subphase containing CS and Ca^2+^ ions (Fig. 2c) at physiologically relevant salt- and sub-micromolar analyte concentrations [39]. During this interaction, the structural changes of the DPPC monolayer were monitored by VSFG spectroscopy. It was found that while the alkyl chains of lipids remain almost unchanged and the surface pressure decreased only few millinewtons per meter, the phosphate groups and the surrounding interfacial water molecules are highly affected by CS (see Fig. 2c). These results led to the conclusion that long CS chains form an organized layer below the DPPC monolayer, at which the CS aligns into a chiral secondary structure, most probably a helical coil [39]. The folding of GAG chains into helical segments was shown previously in solution, but has so far not been demonstrated at interfaces. In the future, with the advent of VSFG spectroscopy, more detailed information about the organization and the structure of GAG chains at model or cell membranes will be possible to be recovered.

While most traditional surface-sensitive techniques require high vacuum or surface selectivity via introducing inorganic interfaces, e.g., plasmonic nanoparticles, the VSFG technique can be directly applied to probe heterogeneous interfaces in situ and real time. Furthermore, such molecular structural information at bio-interfaces cannot be provided by using any other methods; therefore, VSFG spectroscopy will be one of the more powerful techniques to study heterogeneous bio-interfaces.

## Outlook

Until now, spectroscopic experiments with model cell membranes were predominantly focused on proteins and lipids. However, when complex biological interfaces of true physiological relevance are addressed, it is crucial to also include GAGs in the analysis. They are highly abundant at biological membranes and play a crucial role in essential processes at the interface. Currently, technologies to produce homogeneous GAG chains with identical sulfation patterns are emerging, which for the first time will enable a truly systematic assessment of the structure-function relationship of GAGs.

The effect of GAGs on the lipid membrane can be seen from two distinct points of view: (i) the primary effect is when the GAG chain directly interacts with the lipid head groups, which can lead to changes in the conformational order and membrane fluidity, while (ii) the secondary effect is observed during the interaction of proteins with the lipid membrane; here, the interaction can be drastically changed, *e.g.*, by the electrostatic attachment of a polyanion.

GAG-lipid interactions are currently understudied; however, once these fundamental aspects are better understood, the knowledge on the role and structure of GAGs at the cell membrane and the ECM will grow exponentially. Currently, it is not understood how and to which extent GAG chains interact with the lipid membrane. This is further complicated when considering structural aspects. Even though an interaction between GAGs and lipids is expected, it is crucial to elucidate if it is functional or random, and whether a local enrichment of GAGs can be observed in regions where certain lipids are more abundant. Moreover, the interaction of the cell membrane with long GAG chains will likely influence the fluidity of the membrane and influence the uptake of certain cargo from the extracellular space. Changes in the membrane fluidity in close proximity of membrane-associated PGs can have further effect on cellular uptake or signaling. These are just a few examples, which once resolved will help to unravel the detailed molecular processes at the membrane-ECM interface.

Vibrational spectroscopy is a promising analytical tool to study GAG-membrane interactions. This especially applies to techniques that are based on nonlinear optical processes, as their selection rules can provide further benefits, *e.g.*, vibrational information of enriched molecules in a three-dimensional volume or high surface selectivity. With the recent advances in laser technology, broadband VSFG spectroscopy at a laser repetition rate of ≫ 10 kHz has become a biocompatible and user-friendly technique allowing fast data acquisition even at sub-monolayer sensitivity. A range of experiments with increasing complexity of the model membrane will for example provide a better understanding of the dynamics and interactions at the cell membrane. Experiments addressing the structure and interaction of GAGs at the cell membrane will reveal the direct microenvironment of membrane PGs and how their GAG chains affect the fluidity of the cell membrane. Furthermore, these experiments will help to elucidate the role of the three-dimensional structure of the GAG chains on their interactions. However, systematic experiments of GAG-lipid interactions will require a suitable model system. Once the model system is optimized, highly homogeneous, long GAG chains can lead to a deeper understanding of the role of GAGs at the cell membrane-extracellular matrix interface.
